# Composition and applications of focus libraries to phenotypic assays

**DOI:** 10.3389/fphar.2014.00164

**Published:** 2014-07-24

**Authors:** Anne Mai Wassermann, Luiz M. Camargo, Douglas S. Auld

**Affiliations:** Center for Proteomic Chemistry, Novartis Institutes for Biomedical ResearchCambridge, MA, USA

**Keywords:** phenotypic assays, chemical libraries, high-throughput screening, chemical probes, focused library

## Abstract

The wealth of bioactivity information now available on low-molecular weight compounds has enabled a paradigm shift in chemical biology and early phase drug discovery efforts. Traditionally chemical libraries have been most commonly employed in screening approaches where a bioassay is used to characterize a chemical library in a random search for active samples. However, robust curating of bioassay data, establishment of ontologies enabling mining of large chemical biology datasets, and a wealth of public chemical biology information has made possible the establishment of highly annotated compound collections. Such annotated chemical libraries can now be used to build a pathway/target hypothesis and have led to a new view where chemical libraries are used to characterize a bioassay. In this article we discuss the types of compounds in these annotated libraries composed of tools, probes, and drugs. As well, we provide rationale and a few examples for how such libraries can enable phenotypic/forward chemical genomic approaches. As with any approach, there are several pitfalls that need to be considered and we also outline some strategies to avoid these.

## Introduction

Chemical libraries employed for drug discovery ideally contain biologically active chemical scaffolds which are synthetically tractable. Both historically and today compounds are chosen to populate chemical libraries based on ease of synthesis and availability and over time a wealth of information on the bioactivity of these libraries has been collected. For instance, the original compound collections of companies such as Ciba Geigy and Bayer arose out of the dye industry. Repurposing dyes for drug development was a common practice and successful in identifying the first chemotherapeutics (Hager, 2006). Further exploration of dyes composed of quinazolone-3-oxides led to the chance discovery of chlordiazepoxide (Librium®, an anticonvulsant and anxiolytic drug) as one of the first benzodiazepines (Sternbach, 1979). As more high-throughput chemistry approaches became available, compound libraries were designed based on biologically active pharmacophores such as 1,4-benzodiazepin-2-ones and purines providing libraries that are broadly active against a variety of receptors and target classes (Guo and Hobbs, 2003; Dolle et al., 2006). In 1988 Evans and co-workers at Merck coined the term “privileged” structure to recognize the high bioactivity of such compounds and assembling libraries based on privileged scaffolds represents an early use of biological compound annotation to design biology-orientated chemical libraries (Evans et al., 1988). Another example of exploring annotated compound information which has been now applied is the so-called “rule of 5” (Ro5). Through examining route of administration data pertaining to known drugs available from the World Drug Index (WDI), the physicochemical characteristics of well-absorbed drugs were defined based on four parameters: molecular weight (MW), lipophilicity (measured by the partition coefficient logP), hydrogen bond donors, and hydrogen bond acceptors. The Ro5 is based on the fact that all values are multiples of 5 and led to the term “drug-likeness” when characterizing and designing compounds and libraries.

Modern libraries are typically a collection of historical archives, contributions from drug discovery programs (which may add thousands of related analogs as well as clinical candidates), and commercial sources that can include both purified natural products and combinatorial collections. Natural product inspired libraries (Basu et al., 2011; Zimmermann et al., 2013), or methods such as diversity oriented synthesis (Nielsen and Schreiber, 2008) which attempt to expand the composition of current libraries to new areas of chemical property space have also been pursued. At Novartis, fractionated natural product extracts continue to play a key role in discovery efforts. Alternatively, combinatorial collections have been constructed where thousands of slight variations are made around a central scaffold in an attempt to densely cover structure-activity relationships (SAR) within a narrow region of chemical property space (Sanchez-Martin et al., 2004). Overtime, a significant amount of bioassay data has accumulated on compound libraries leading to both private and public databases that provide an archeological footprint of past discovery efforts. These databases can be configured to provide a rich source of small molecule bioactivity data to drive future discovery efforts. Here we provide examples of how this information can be used to design libraries composed of chemical tools, probes, and drugs. Definitions and examples are given for various types of compounds and libraries. We also outline some of the issues involved with applying annotated compound libraries.

## Categories of compounds employed in focus libraries

**Figure 1:**
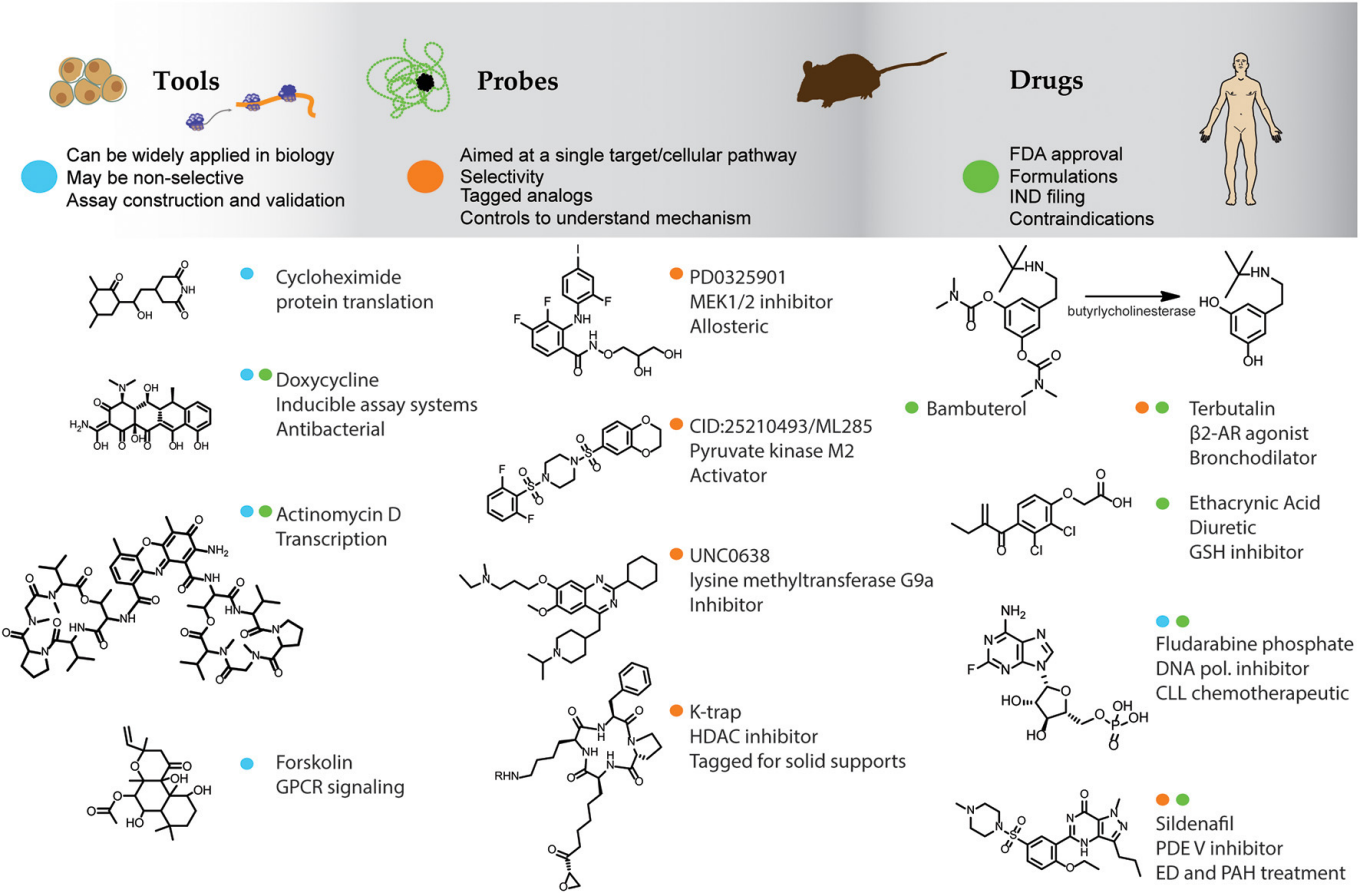
**Small molecules as tools, probes, and drugs.** The top bar lists some general features of compounds used as tools, probes, and drugs and example compounds are listed underneath. Color dots designate the primary use of the compounds as a tool (blue), probe (orange), or drug (green). β_2_-AR, β_2_-adrenergic receptor; GSH, glutathione S-transferase; CLL, chronic lymphocytic leukemia; ED, erectile dysfunction; PAH, pulmonary arterial hypertension.

Compounds that have been applied to biological systems can be classified as tools, probes, or drugs (Figure 1). Tool compounds can be broadly applied to understand general biological mechanisms. Examples include cycloheximide (Figure 1), a natural product adopted by cell biologists as a means to study translational mechanisms. Cycloheximide is too toxic for *in vivo* studies but is widely applied to *in vitro* cell-based assays. Some compounds such as Actinomycin D (Figure 1), a natural product from bacteria that inhibits the function of RNA polymerase, is both a chemotherapeutic and a tool compound used to test for transcriptional mechanisms. Similarly doxycycline (Figure 1) is used both as an antibacterial drug and as a tool to develop inducible cell-based assays using the tetracycline repressor system (Gossen and Bujard, 1992). The natural product forskolin (Figure 1), and its water-soluble analogs (Laurenza et al., 1987), stimulates adenylate cyclase serving as a critical tool compound to study and develop assays for G_αi_/G_αs_ coupled GPCRs. Such compounds have an essential role in the cell-biologist's tool box.

Employing compounds to probe complex biological pathways has its roots in the discipline of bioorganic chemistry. Konard Bloch's use of deuterated acetate to investigate the biosynthesis of cholesterol, described in 1942 (Bloch and Rittenberg, 1942), could be attributed to an early use of a chemical probe. However, modern chemical probes have primarily been discovered through high-throughput screening (HTS) efforts. Chemical probes as applied to *in vitro* assay systems may have a more limited application compared to tool compounds as these are specifically designed to modulate an isolated target protein or signaling pathway. General guidelines for what constitutes an optimal chemical probe will depend on the application and context of interest but includes chemical properties related to stability, solubility, availability, and cell permeability, as well as the potency and selectivity of the compound (Frye, 2010; Workman and Collins, 2010). Some of the first initiatives in probe discovery were at the Harvard Institute of Chemistry and Cell Biology (ICCB) founded by Mitchison and Schreiber in 1998 where chemical approaches and biological disciplines were merged and organized into a new area of research (Hager, 2006; Huang et al., 2011). In 2003 the US National Institutes of Health (NIH) started the Molecular Libraries Initiative (MLI) to provide industrial-scale HTS technologies and chemical probes for basic research (Lipinski et al., 1997; Klekota and Roth, 2008). Through this effort many young investigators have been successfully trained in the methods of compound discovery and >300 reports describing new chemical probes have been published^1^. There are now several initiatives in chemical biology in both the United States and Europe. Guidelines for chemical probe designation have been suggested^2^ which includes the demonstration of SARs wherein both active and inactive analogs of a chemical series are identified.

Some example chemical probes are shown in Figure 1. Trapoxin, isolated from the fungus *Helicoma ambiens*, is a compound that naturally contains an epoxide group and was initially characterized as a cell cycle inhibitor capable of inhibiting histone deacetylation in cell lysates. The trapoxin analog K-trap (Figure 1; Taunton et al., 1996a) allowed coupling to a solid support which identified the molecular target as a protein with high homology to transcriptional repressors (Taunton et al., 1996b) and resulted in the characterization of the class of enzymes known as histone deacetylases (HDACs). The allosteric MEK1/2 inhibitor PD0325901 (Figure 1) shows good selectivity against other protein kinases and has been used as probe for this kinase in both *in vitro* and *in vivo* assays (Ohren et al., 2004; Bain et al., 2007). One of the grand challenges of chemical biology is to provide chemical probes for every protein expressed from all human genes. Some chemical probes have been successfully developed toward new target classes such as the lysine methyltransferase inhibitor UNC0638 (Figure 1; Vedadi et al., 2011), providing a means to explore the function of this enzyme in model systems. In another recent example, chemical probes have been identified with novel mechanisms such as ML285 (Figure 1) which is an activator of the M2 isoform of pyruvate kinase, expressed in cancer cells, and has been used as probe to study the Warburg effect in cancer cells and animal models (Brimacombe et al., 2010; Anastasiou et al., 2011).

Drugs are the most widely recognized small molecules due to their beneficial pharmacological effects. Only a few thousand compounds have been approved as drugs by the FDA since 1950 (Munos, 2009, 2013). Drugs are indeed the exception in small molecule research largely due to the strict requirements of bioavailability, low toxicity, and metabolic stability that constrains the properties of compounds intended to be approved as drugs (Figure 1). The physicochemical properties of drugs include modifications aimed at improving the absorption, distribution, metabolism, and excretion (ADME) properties for *in vivo* use but such properties are often irrelevant or even a hindrance to chemical probes aimed at *in vitro* studies. ADME properties are often engineered into drugs at the expense of potency, a property more important to chemical probes, and therefore drugs are sometimes not useful as chemical probes. In the extreme example of a prodrug, the compound is inert until placed *in vivo* where metabolism releases the active component. For example, the drug Bambuterol (Figure 1) has been protected with carbamate moieties to provide a slow-acting form of the β2-adrenoreceptor agonist Terbutalin through the action of butyrlycholinesterase (Huttunen et al., 2011). Ethacrynic acid (Figure 1) is a drug which acts as a diuretic through inhibition of Na-K-Cl cotransporters and contains a Michael acceptor which inhibits the enzyme glutathione-S-transferase via a covalent mechanism. Such reactive compounds may be less useful as chemical probes due to instability and promiscuity in an *in vitro* setting. On the other hand, drugs such as the chemotherapeutic Fludarabine phosphate (Figure 1), acting through general inhibition of DNA synthesis, may serve as useful tool compounds while drugs such as Sildenafil (Figure 1) with a specific target (phosphodiesterase; PDE) can be a useful chemical probe to test for the function of PDEs in cell based assay systems. Other drugs cannot be used as tools or probes because they have an undefined or complex mechanism of action. For example, Modafinil is a drug marketed for wakefulness disorders discovered in the 1970s for which the molecular mechanism of action remains unknown. As well, antipsychotics often show a complex polypharmacology that is required for efficacy but their mechanism is not well understood (Roth et al., 2004; Garcia-Serna and Mestres, 2011), this is discussed further below. Therefore, while it may be useful to study the underlying mechanisms of drugs using chemical biology techniques, efforts employing certain drugs to help understand the mechanisms underlying biological activity could be challenging requiring the combination of chemical and systems biology approaches to fully leverage such compounds.

## Approaches and rationale for library design and applications to phenotypic assays

The use of annotated compound collections to enhance the understanding of the activity arising from phenotypic assays has been practiced for more than 10 years (Root et al., 2003). Currently, such focused libraries are playing an increasing role when implementing either target-based or cell-based assay approaches due to the desire to investigate new biology and mechanisms. Library design approaches can be thought of in three ways (Box 1) and each of these will be described below with an emphasis on applications to cell-based and phenotypic assays.

Box 1 **Target-based vs. phenotypic screening libraries.**

**Target-oriented libraries:** A focused library is built around a target or target family from known pharmacophores or privileged scaffolds. These scaffolds are derived from historical experimental bioactivity data as well as ligand- or structure-based virtual screening approaches.

**Phenotypic-oriented libraries:** A maximally biologically or chemically diverse library is assembled. Special emphasis might be given to physicochemical properties of compounds to optimize cell permeability and solubility.

**Hypothesis-driven phenotypic libraries:** Based on the knowledge about the biological model/pathway under study, bioinformatics and cheminformatics resources are leveraged to link diseases, molecular processes, pathways, and functions to small molecules. Resources are listed in Table S1.

### Target-oriented libraries

In the era of target-based screening, much effort was dedicated to the design of target-focused libraries, i.e., screening collections of compounds specifically tailored to modulate a given target or target family (Harris et al., 2011). The aim of these library design efforts was to increase hit rates and cost-benefit ratios by screening compounds with an *a priori* higher likelihood of being active against the target. Depending on the amount of structural and ligand information available for a target (or family) of interest, various approaches for the design of such target-focused libraries were developed and successfully applied in the past. For example, if crystallographic structures of the target (family) of interest are available, docking algorithms can be used to choose compounds with good complementarity in electrostatics and shape to the protein binding pocket. This strategy has been frequently pursued for kinases (Lowrie et al., 2004; Orry et al., 2006). By contrast, if a large number of known active ligands can be retrieved, ligand-based design methods using molecular fingerprints or pharmacophore models might be the method of choice, as successfully applied to GPCRs and ligand-gated ion channels (Lowrie et al., 2004; Harris et al., 2011). For target families such as GPCRs, in which rich active chemical matter is available for certain receptor classes, redesigning known ligands to show a desired activity profile can become an engineering exercise readily handled by sophisticated computer algorithms (Besnard et al., 2012). In many cases, target-focused libraries cover only limited structural diversity and are based around a few core (or privileged) scaffolds that are differently substituted at a number of attachment points.

### Libraries for phenotypic screens: chemical and biological diversity

**Figure 2:**
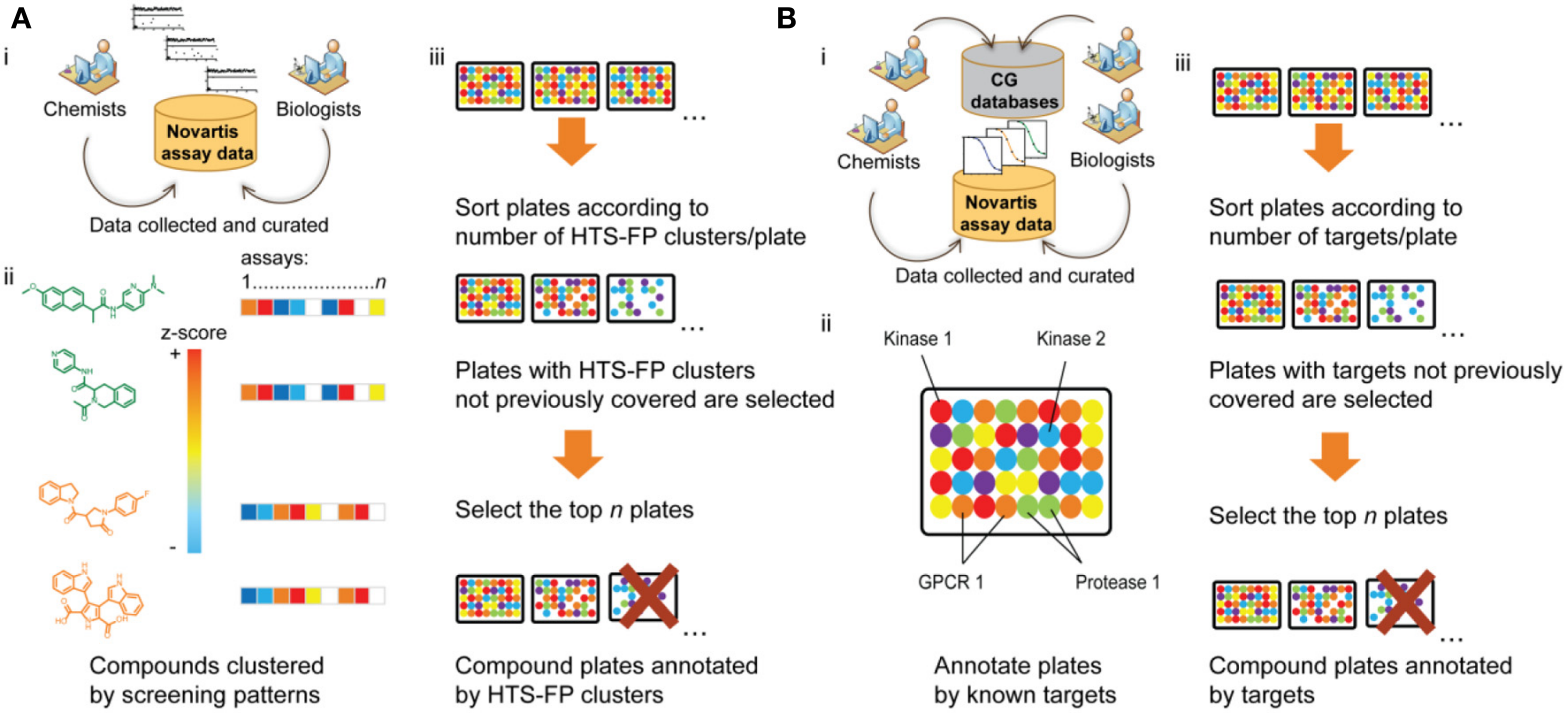
**Shown are two different approaches developed at Novartis for the design of biodiverse compound libraries from a pre-plated screening deck. (A)** For each compound in the Novartis screening collection, primary assay data from more than 200 HTS campaigns are collected, standardized in form of *z*-scores, and stored in a minable activity pattern (HTS-FP). This requires construction of a robust bioassay ontology and curation of compound and assay information. Then compounds are clustered by their activity patterns and screening plates are ranked in descending order of the number of different bioactivity clusters covered by the compounds contained in each plate. The more clusters are found on a plate, the more dissimilar the plated compounds are with respect to their bioactivity patterns and the higher the biodiversity of the plate. Therefore, plates from the top of the ranking are selected for screening (unless their cluster composition is redundant with higher ranked plates). **(B)** For each compound in the Novartis screening collection, concentration-response data is extracted from curated internal and external structure-activity databases. Compounds on a plate are annotated with their known targets and the biodiversity of a plate is measured by the number of different protein targets known to be modulated by the compounds on the plate. After ranking of the plates according to their biodiversity, the selection process is analogous to **(A)**.

Historically, the majority of drugs have been discovered through phenotypic drug discovery approaches where one advanced *in vivo* activity in a manner agnostic of the molecular mechanism of action. Both target-based and phenotypic drug discovery approaches are practiced today with inherent advantages and disadvantages (Swinney and Anthony, 2011). Typical modern phenotypic assays include cell proliferation or selective growth inhibition assays oftentimes applied within oncology or infectious disease areas. Cell-based pathway–based approaches applied to chemical library screening efforts include the wide use of engineered cell lines in a reporter-gene assay (RGA), intracellular sensors, or high content screening approaches (Inglese et al., 2007; Inglese and Auld, 2009). In many of these cases, a screening library covering a broad spectrum of targets and molecular processes is generally most promising. For many years, the predominant paradigm for the assembly of such a library was to select a chemically maximally diverse compound set using diversity measures based on molecular scaffolds (Krier et al., 2006), physicochemical properties, 2D and 3D chemical structure (Matter, 1997), or pharmacophore descriptors (Mason et al., 2001). Irrespective of the specific diversity method used, all these library design strategies followed the belief that chemical diversity ultimately translates to biological diversity and that a chemically diverse screening library should hence be a suitable starting point for many different drug discovery projects. However, in many cases, these chemically diverse sets were not truly randomly chosen from chemical space given the widely accepted assumption in medicinal chemistry that not all parts of chemical space are biologically active or relevant. For example, Hert et al. (2009) have argued that, given the size of chemical space, the odds of finding a hit in a random diverse selection of ~1 million compounds seem rather negligible. They explain the success of HTS against these odds by the biogenic bias of screening libraries, i.e., their higher than average synthetic small molecule similarity to natural products and metabolites. By default, these naturally occurring molecules interact with biological systems and can therefore be viewed as representatives of biologically active chemical space. The idea of tailoring screening libraries toward biologically relevant chemical space is taken to the next level by strategies that directly integrate the known biology of compounds into screening set design by maximizing the known biodiversity instead of the chemical diversity of screening collections. These biodiversity methods have been made possible by the wealth of screening and small molecule bioactivity data that has been released over recent years in both corporate and public domains. For example, at Novartis, two different approaches for the selection of biodiverse screening sets have been developed (Figure 2).

The first approach uses primary activity data from more than 200 biochemical and cellular HTS datasets that have been performed at Novartis over the past decade (Petrone et al., 2012). In this approach, a screening pattern (so called HTS fingerprints; HTS-FP) capturing the activity for all molecules across this large assay panel is derived (Figure 2A). With this information in hand, the Novartis pre-plated compound archive is prioritized so that compound plate sets are assembled to represent as many distinct screening patterns as possible (Petrone et al., 2012). A similar route was taken in the assembly of a mechanistic diversity set from compounds tested in the NCI60 cancer cell line panel that was selected to cover a broad range of growth inhibition patterns (Monga and Sausville, 2002)^3^.

The second biodiversity selection approach employed at Novartis (Petrone et al., 2013) uses bioactivity data found in internal and external bioactivity databases (see Table S1). All pre-plated compounds are annotated with their protein targets from concentration-response measurements using a defined activity threshold (typically AC_50_ ≤ 5 μM). After curation of compound-target interactions, the number of different targets modulated by the compounds on a plate is counted (Figure 2B). Constraints on compound sets can be made so that plates with a high coverage of different targets are prioritized for screening while plates with redundant target space coverage are removed from the set.

Interestingly, benchmark calculations comparing the efficiency of chemical and biological diversity methods for plate-based selection of 250,000 small molecules concluded that chemical diversity is a necessary, but not sufficient condition for the success of a screening library (Petrone et al., 2013). By contrast, compound sets that were both chemically and biologically diverse achieved the highest hit rates across a number of biochemical and cellular screens (Petrone et al., 2013). In particular, compound libraries selected based on known compound-target interactions extracted from bioactivity databases achieved the best results. It should be noted that different assumptions are made for the success of such a biodiverse library in biochemical and phenotypic screens. The success of a biodiverse library for a biochemical screen can be explained by the hypothesis that compounds that were previously found to be active are more “hit-like” than other molecules and are therefore more likely to show bioactivity in future screens against novel targets. In this case, the frequently observed polypharmacology of small molecules is exploited. For phenotypic screens, a second target activity is not even required for a positive activity outcome but the already known target activity could lead to the desired phenotypic effect. In fact, screening of a biologically annotated compound library can help to overcome one of the major bottlenecks in drug discovery: target elucidation of a hit list from a phenotypic screening campaign. By using the known annotations of screened compounds, targets that are enriched among the active molecules can be inferred. Likewise, the target annotation of a small molecule can be used to link a compound to a molecular process or pathway in which this target plays a role and processes/pathways that are overrepresented for the hit set can be detected. This enrichment analysis leads to a better mechanistic understanding of the phenotypic screen and can directly be applied in hit triaging to select compounds with a desirable mechanism of action (MoA) for follow-up assays while removing compounds with an unwanted MoA, e.g., the inhibition of a target that is known to show broad cell cytotoxicity. Such an understanding can help anticipate assays in the discovery flow chart so that the appropriate counterscreens and secondary assays are made available to follow-up active compounds.

### Hypothesis-driven phenotypic screening libraries

Earlier in this article biologically and chemically diverse screening sets were described as adequate libraries for phenotypic screening if no assumption about the molecular processes captured by the phenotypic screen is made. However, if prior knowledge about the biological model under study exists, e.g., through a pilot screen with a compound library or literature mining, it is often advisable to customize the screening library. Data repositories such as OMIM (Amberger et al., 2011), Gene Ontology (Blake et al., 2013), GeneGo Metabase, and KEGG Pathways (Kanehisa et al., 2014) can be used to establish connections between diseases, molecular functions, processes, pathways, and involved protein targets. In turn, public and commercial bioactivity databases such as ChEMBL (Gaulton et al., 2012), DrugBank (Law et al., 2014), and PubChem BioAssays (Wang et al., 2014) are a rich resource for linking these presumably relevant targets to small molecules thereby enabling hypothesis-driven compound cherry-picking for phenotypic screening. An overview about bioinformatics and cheminformatics resources that can be exploited for the design of focused phenotypic screening libraries is given in Table S1.

Although biologically annotating small molecule collections is simple in theory and concept and the resources mentioned in Table S1 are easily accessed, one has to consider several difficulties which are often encountered when accessing these databases. These issues mainly stem from errors in the databases and the difficulty of obtaining unified representations of compound structures and protein targets used by different data repositories. Multiple studies published by Kramer and colleagues have investigated sources of compound bioactivity database errors and the experimental uncertainty associated with published *K*_*i*_ and IC_50_ values (Kramer and Lewis, 2012; Kramer et al., 2012; Kalliokoski et al., 2013). Kramer and Lewis (2012) discovered copy-and-paste errors introduced upon transcription of activity data from original literature to databases include incorrectly reported units (e.g., a protein-target interaction is reported with a potency of 30 nM in the database although the underlying publication reported a potency of 30 μM) and a wrong extraction of the target protein (e.g., confusion of different receptor subtypes or omission of the receptor subtype information in the database). Moreover, compound structures are often incorrectly assigned, with a predominance of stereochemistry errors. As well, compound-target interactions that were formally correctly extracted from the literature cannot all be considered equally reliable. For example, determination of IC_50_ values depends on the assay conditions and a compound reported as highly active against a target by one laboratory may show weak activity in another laboratory depending on the choice of substrate, concentration, and other parameters that were used in the experimental design. At least for enzyme assays there are initiatives in place to help standardize the reporting of enzyme activity and inhibition data such as STRENDA (Standards for Reporting Enzymology Data) (Auld and Acker, 2014; Tipton et al., 2014). However, cell-based assays tailored toward a specific protein target are often more difficult to interpret and it is always possible that the observed activity results from an off-target process. Nevertheless, in a database, the assay result is often reported as the activity of the compound against one defined protein target although the binding event has not yet been established. Therefore, detailed knowledge of the assay protocol is often necessary to judge the quality of the reported compound activity. However, assay descriptions are often not provided by databases or, if they are available, are in free-text format, which is difficult to interpret and mine for large-scale annotations of compound collections. An initiative that tries to address this problem is the BARD^4^ (BioAssay Research Database) project that uses standardized terms to describe experimental contexts and corresponding result data (De Souza et al., 2014). The ChEMBL database uses scores that are created during the manual curation process of the data and which reflect the confidence that the correct protein target has been assigned for the reported assay. Robustly curated compound activity databases, i.e., data quality and not necessarily data quantity, will be crucial for mining and interpretation of phenotypic screening results. Also, users of databases should be wary of the wealth of bioactivity data that is available and critically consider these potential pitfalls and the quality level of the reported results.

Another challenge faced by researchers using different sources of biological annotation is data integration. As detailed in Table S1, a multitude of informative data repositories exist, however, linking compounds or protein targets in one database to another is a non-trivial task due to the usage of different compound and target representations. For example, small molecules might be registered in a database by SMILES (Weininger, 1988), InChI keys (Heller et al., 2013), or CAS numbers and proteins by UNIPROT (Uniprot, 2010), Ensembl (Flicek et al., 2011), or Entrez gene (Maglott et al., 2011) identifiers. Therefore, data from different repositories needs to be unified to enable cross-links and a full leverage of available data. Whereas solutions for data integration have been found by pharmaceutical companies that often combine data from various internal and external sources in one data warehouse, data access is typically more difficult for smaller academic labs that do not have the same resources for data management and less informatics support.

Not only biological annotations but also the chemical structures of hits can be exploited to expand around results from a library screen employing a phenotypic assay. Even if the targets of phenotypic screening hits are not known, it has been shown that it is possible to learn physicochemical properties and/or structural features from these active compounds that can be used to develop new compound sets that are likely to show the same phenotype. For example, taking data from a yeast growth inhibition screen, Wallace et al. (2011) trained a Naïve Bayes model to distinguish substructures found within active and inactive compound sets. Focused compound sets chosen with this model were 2 to 4-fold enriched with phenotype-inducing compounds. Interestingly, growth inhibitory effects of these compounds were not restricted to yeast as the model was successfully applied to enrich focus libraries with active compounds across diverse model organisms.

Hypothesis-driven focused sets aimed at a particular phenotype can be very enabling to phenotypic assays that often involve difficult protocols and precious cell samples such as co-culture and 3D-culture systems employing primary cells. With increased effort to move to more disease relevant phenotypic assays employing the use of precious biological samples, the design of focus libraries to efficiently screen these assays becomes paramount. The results from focus library testing can enable a hypothesis related to the underlying biological mechanism of the phenotypic response and anticipation of the necessary secondary assays required to test the hypothesis.

### Example tool compound and probe libraries

The types of compounds described in the first section of this review can be employed to construct focus libraries which are applied to understand and characterize biological assays. Over the past years, many efforts have been geared toward the design of explicit tool compound or probe libraries, i.e., small molecule libraries assembled to perturb and investigate pathways and signaling cascades in phenotypic assays. These libraries are often tested in a pilot screen before running an assay on a larger screening deck, either with the purpose to validate the assay (i.e., to test whether compounds that are expected to be active based on their known MoA show up as hits) or to gain a better mechanistic understanding of the activity arising from phenotypic assays. This means that, through their design, these compound sets enable MoA hypothesis formulations and the identification of targets involved in a biological model. Typically, these libraries consist of <10,000 low molecular weight compounds covering diverse biological activities. If possible care is taken that multiple compounds modulate the same target: if all compounds with a common MoA evoke the same phenotypic effect, confidence is increased that this phenotypic activity is related to their shared target and not the result of an unknown off-target effect. One example of such a compound set is the Library of Pharmacologically Active Compounds^5^ (LOPAC) marketed by Sigma Aldrich. This library currently containing 1,280 compounds is largely composed of drugs and therefore has some limitations for MoA analysis as noted previously including an over-representation of well explored target classes such as GPCRs and protein kinases. However, the LOPAC collection was recently used to study lymphangiogenetic sprouting in a 3D assay, identifying MEK 1/2 inhibitors and also the drug class of statins that inhibit the enzyme HMG-CoA reductase as blockers of lymphangiogenesis (Schulz et al., 2012). Similarly, the Target Discovery Institute (TDI) at the University of Oxford is currently constructing the TDI Small Molecular Probe Library which is described as a collection of 3000–4000 compounds “chosen to explore complex diseases pathways and to assist in the characterization of disease targets”^6^. GlaxoSmithKline (GSK) has assembled a tool box of about 6000 compounds termed Biologically Diverse Compound Set (BDCS) that targets 736 unique proteins (Liu et al., 2014). Up to 10 maximally selective compounds from different chemical series are included for each target. The compound set was tested for suppression or enhancement of histone-3 K27 tri-methylation (H3K27me3). In addition to compounds targeting methyltransferases or demethylases, a number of compounds without direct activities against these targets were identified and suggested to be upstream pathway regulators (Liu et al., 2014). Mapping the compounds' protein targets back to pathways led to the observation that the ErbB2-ErB3 signaling pathway was significantly overrepresented among the targets of active compounds, implying an involvement of this pathway in H3K27me3 regulation. Many target-oriented libraries are available for historical drug classes such as GPCRs and kinases. The kinase inhibitor set from GSK (Dranchak et al., 2013) contains over 300 kinase inhibitors and can be used to test an assay's sensitivity/response to particular kinases and build pathway hypotheses. All focus libraries of bioactive compounds are best screened in a concentration-response mode as these libraries will often have high hit rates (>10%). Titration-based screening of pilot libraries in a quantitative HTS (qHTS) format can provide robust potency and efficacy data to construct target/pathway hypothesis as well as typical assay performance statistics such as Z-factors (Inglese et al., 2006; Zhang et al., 2013).

### Considerations on compound properties

One aspect that is not further discussed in this article is that phenotypic screening also puts constraints on the physicochemical properties of small molecules. In many cases, high cellular permeability is crucial for a compound to be active in a phenotypic screen. Therefore, phenotypic screening libraries are optimally designed with consideration of compound solubility and cell penetration, as for example the phenotypic screening set by Asinex^7^. Furthermore, compound structures with known cytotoxic features were removed from this screening collection.

## Analysis and interpretation of annotated chemical library data

For any assay result one needs to investigate the origins of compound activity, or lack thereof, with a consideration of compound properties as possible factors that may confound the observed results. Research aimed at understanding assay artifacts has led to an understanding of common chemotypes that can skew the results arising from certain assay technologies (Thorne et al., 2010). A number of profile studies have been performed to determine the types of compounds that form colloidal aggregates (Feng et al., 2007; Owen et al., 2014), interfere with common fluorophores through compound autofluorescence (Simeonov et al., 2008), show pan assay interference (Baell and Holloway, 2010), interfere through redox mechanisms (Johnston et al., 2008), and those that inhibit and stabilize reporter gene enzymes in cells (Thorne et al., 2012; Ho et al., 2013). With this in mind, focus sets can be designed to measure an assay's susceptibility to artifacts and this knowledge can then be used to anticipate the necessary counterscreens. For example, the GSK kinase inhibitor set has been screened against firefly and *Renilla reniformis* luciferases because these are common reporter enzymes used to construct assays (Dranchak et al., 2013). Such an analysis should always be performed before using chemical library screening data for bioactivity analysis.

**Figure 3:**
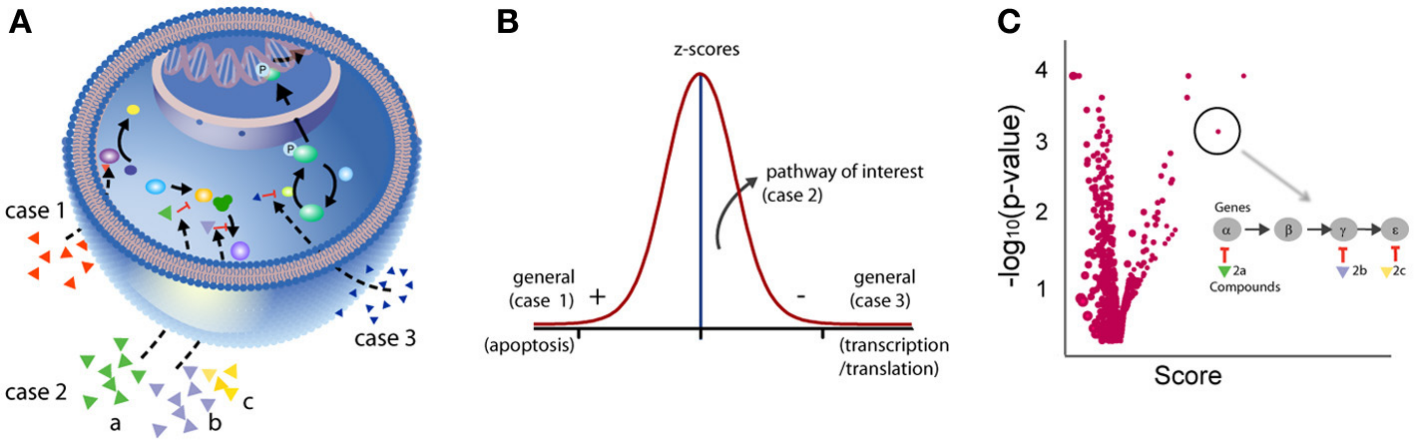
**Analysis of the entire bioactivity dataset.** Examining biological annotations for the entire dataset including activity outside the extremes can rescue weak activity. **(A)** Depicted are three cases where compounds modulate cell signaling. In case 1, a compound shows high activity through, for example, activation of apoptosis. In case 2, three compounds are annotated to targets known to function in the same signaling pathway but each compound shows weak activity in the assay. In case 3, a compound shows strong activity in the assay acting through inhibition of general mechanisms such as transcription/translation. **(B)** While compounds in case 1 and 3 are easily captured using standard statistical thresholds, compounds in case 2 may only be rescued by examining the entire dataset and considering the target annotations in the context of biological pathway analysis. **(C)** Plot representing the aggregate effect of compounds perturbing a pathway to identify weakly active compounds (case 2). Each circle on the plot represents a pathway. The x-axis represents the aggregate effect of compounds hitting targets from the same pathway. For example, case 2 is depicted as having three compounds that individually show weak activity but are annotated as operating in the same pathway. The likelihood of getting such a score by chance, given the number of genes/proteins, is given on the y-axis (given as −log_10_, e.g., *p* = 0.01, 2). Using such an approach, relevant biological processes with subtle effects can be identified.

In addition to taking compound interference and chemical properties into consideration during the analysis of phenotypic screens, it is also important to understand how such focus library screens can be better interpreted. For example, unlike random screening, the expected hit rate in a focus set should be high as by design the compounds have been selected based on some prior knowledge (e.g., they target proteins in a pathway involved in the disease or phenotype of interest). Secondly, the definition of a “hit” is less clear as phenotypic screens measure a biological outcome that can result from the perturbation of different biological processes in the cell with varying degrees of potency. In biochemical target-based assay systems, high potency is a reasonable way to select actives. Conversely, when using cell-based/phenotypic assays, this approach, although still useful, can be problematic. This is because when screening a large collection of compounds that target different cellular processes, one may observe a large effect on the phenotype by compounds that simply target generic cellular mechanisms (e.g., cell-health related mechanisms, Figure 3) which, although these have genuine biological activity, are less interesting or non-specific. For example, it may be no surprise that HDAC inhibitors score in an assay that measures transcription (e.g., an RGA) or that proteasome inhibitors would have a strong effect on assays that measure protein production. Additionally, focusing on the strongest hits may result in missing compounds that effect specific nodes of a relevant pathway but have smaller effects in the assay. The smaller effect of such compounds can be attributed to several reasons: (1) position of the target on the pathway where compensatory mechanisms and pathway cross-talk may result in a minor perturbation of the phenotype; (2) lack of uniformity in the specificity and potency of compounds; (3) lack of uniform chemical properties across the library (cell permeability; binding kinetics), and (4) lack of congruence between the time required to observe a phenotypic effect by perturbing a given target vis-à-vis the duration of the screen. Therefore, the interpretation of phenotypic screens should take full advantage of prior biological knowledge, such as target and pathways, to facilitate analysis.

Compounds that modulate upstream nodes in a pathway may have much smaller effects on the assay signal but these weak hits could be rescued by considering if their target annotations place them in the same pathway (Figure 3). In this way the entire dataset can be considered independent of potency or activity threshold values and weak activity can be considered if this activity significantly clusters within specific pathways (Figure 3) (Subramanian et al., 2005; Levine et al., 2006). In other words, the aggregate effect on a biological pathway level is of highest interest as opposed to individual compound activities. Such an analysis enables: (1) identification of mechanisms of interest: known or unknown pathways; (2) identification of new biological endpoints to help validate the observation (e.g., a pharmacodynamic marker for a given biological process); (3) selection of compounds that would help validate the hypothesis; and (4) rescuing weaker but biologically relevant compounds.

The use of prior biological knowledge is particularly powerful when screening with focused libraries where the MoA of compounds is known. The identification of novel pathways and processes can help establish additional endpoints to be measured in a screening funnel or be used as surrogate readouts—ones that correlate with the phenotype of interest—and that may be more amenable to a large scale HTS campaign thus enabling access to a much larger chemical space. For example, if in a given focused library screen, perturbing the JNK pathway has a significant outcome in the measured phenotype, then PD biomarkers within the JNK pathway can be selected as orthogonal measurements in a screening funnel. Alternatively, such an observation can be used to help validate the screen due to prior knowledge that the JNK pathway is implicated in the biology being measured. Ultimately, the success of any screen depends on the interpretability of the results where the combination of a good compound collection, measuring the right biological endpoints, and application of analytical approaches will increase the chances of identifying biologically relevant leads, particularly if challenges such as polypharmacology and pleiotropic effects of molecules are addressed and taken into consideration.

### Challenges and future directions/opportunities

More and more evidence points to the fact that drugs previously perceived to be selective actually exert their effect through more than one target (Hopkins, 2008; Reddy and Zhang, 2013). Furthermore, with the advent of genomic technologies such as gene expression profiling, it has become evident that even drugs with high target selectivity have pleiotropic effects by modulating diverse downstream signaling events (Lamb, 2007; Dobbelstein and Moll, 2014). Such effects could contribute to efficacy and/or adverse events. This has led to a shift from SAR analysis based on a single endpoint to modeling of multiple target activities (Lopez-Vallejo et al., 2012; Petrone et al., 2012; Medina-Franco et al., 2013) and also to the identification of compound relationships based on their effect on a biological system as measured by gene expression (Lamb, 2007). Therefore, the design and interpretation of focused libraries is shifting to include a combination of computational approaches that merge chemo-informatics, bioinformatics, and systems biology.

Polypharmacology presents a challenge in the interpretation of phenotypic screens because it is not necessarily evident which target is contributing to the effect. Furthermore, screening usually takes place at high concentrations, e.g., 10–20 μM. If the library contains both optimized bioactive molecules and also blunt scaffolds, screening at such high concentrations favors the polypharmacology of the optimized molecules to be in full display making the results hard to interpret. This is another reason that a titration-based screening approach should be used when employing MoA libraries, as mentioned above.

Polypharmacology can be established *a priori* by both experimental and computational approaches. Experimentally, biochemical profiling of target classes can give an idea of the selectivity profile for a given molecule. Similarly, gene expression profiling of compounds can shed light on the processes regulated by the bioactive molecules. Computational approaches to establish polypharmacology of compounds have been developed employing either properties of the ligand or the structure of the targets (Houghten et al., 2006, 2008; Hopkins, 2008; Weill and Rognan, 2009; Renner et al., 2011). In addition, by combining gene expression and protein interaction networks, systems biology based methods are also employed to reverse engineer the upstream targets that best explain the gene expression profile (i.e., pleiotropic effects of a compound; Jaeger et al., 2014). All of this information can then be leveraged to increase the interpretation of the screen as, for example, through pathway-based methods as described above. In addition, the polypharmacology of drugs can be used in the rational design of focused libraries. Because kinases participate in many different signaling pathways, one could design a library containing kinase inhibitors that have distinct and/or different degrees of overlapping polypharmacology to act as sentinels for pathways. If the polypharmacology is known, either through computational or experimental methods, it may then be possible to de-convolute the result into the likely targets/pathways involved in the phenotype. For example, Gujral et al. (2014) identified kinases involved in the regulation of cancer cells by exploiting diverse pharmacology of kinase inhibitors whereas Houghten et al. (2006) have developed de-convoluting methods for compound mixtures in *in vivo* screens.

## Conclusions

The composition and applications of compound libraries is evolving significantly. Large archives containing analogs for known target classes (e.g., kinases, GPCRs), combinatorial collections, purified natural products, and diversity oriented synthesis usually employed in large scale HTS-campaigns can now be optimized by selecting a subset of compounds that maximize both biological and chemical diversity. More recently, largely due to the increased use of complicated phenotypic screens—usually limited in throughput and interpretability—smaller libraries will need to be compiled that leverage prior biological knowledge and the polypharmacology and pleiotropism of the compounds. For example, many *ex vivo* assays such as long-term potentiation or multi-electrode arrays can be employed as phenotypic assays that measure neuronal activity but these assays have extremely low throughput (Thomas et al., 1972; Bliss et al., 2004). Improved strategies to build such libraries coordinated with the development of computational methods to interpret the results are needed.

The ever growing body of orthogonal chemical biology datasets will continue to change our understanding of compound properties and how compounds are selected for focus library development. For example, biological profiling may help identify molecule classes, which although chemically distinct, have a common biological mechanism and provide a means for compound repositioning or an understanding of adverse effects (Lounkine et al., 2012). Similarly, systematic efforts to understand the toxicity of compounds have resulted in large publicly available datasets with noted examples including data sets from Iconix Biosciences (Ganter et al., 2005), the National Institute of Biomedical Innovation (NIBIO, Japan) (Uehara et al., 2010), and ToxCast released from the Environmental Protection Agency (Chen et al., 2012; Kavlock et al., 2012; Knudsen et al., 2013). Optimal employment of these databases requires expert teams of biologists, chemists, and informatics scientists with critical consideration of the source data. However, the combination of historical and ongoing chemical profiling (PubChem, ChembL) with improved annotation and data mining tools (such as BARD) will lead to improved methods to design focused libraries.

### Conflict of interest statement

The authors and editor declare that while they are currently affiliated with Novartis, there has been no conflict of interest during the review and handling of this manuscript. The authors declare that the research was conducted in the absence of any commercial or financial relationships that could be construed as a potential conflict of interest.

## Footnotes

^1^ http://www.ncbi.nlm.nih.gov/books/NBK47352/

^2^ https://commonfund.nih.gov/molecularlibraries/index

^3^ http://www.dtp.nci.nih.gov/branches/dscb/mechanistic_explanation.html

^4^ https://bard.nih.gov/BARD/

^5^ http://www.sigmaaldrich.com/chemistry/drug-discovery/validation-libraries/lopac1280-navigator.html

^6^ http://www.tdi.ox.ac.uk/small-compound-libraries

^7^ http://www.asinex.com/libraries-phenotypic-screening-set12.html
