# Supplementary material for: Composition and applications of focus libraries to phenotypic assays
Source: Front Pharmacol. 2014 Jul 24;5:164. doi: 10.3389/fphar.2014.00164 (PMC4109565; doi:10.3389/fphar.2014.00164)
Supplement: Supplementary file 1 [file Presentation1.PDF]

Supplemental Material
Composition and applications of focus libraries to phenotypic assays

Anne Mai Wassermann, Luiz Miguel Camargo, Douglas S. Auld*

Center for Proteomic Chemistry, Novartis Institutes for Biomedical Research, 250 Massachusetts Avenue, Cambridge, MA, 02139, USA

*Corresponding author: Douglas Auld, Center for Proteomic Chemistry,Novartis Institutes for Biomedical Research, 250 Massachusetts Avenue, Cambridge, MA 02139, USA. E-mail: douglas.auld@novartis.com.

Supplemental Table S1
Cheminformatics and bioinformatics resources for building hypothesis-driven libraries

Resource	Link	Data	Provider	Type
Compound-Target				
BindingDB (Liu et al., 2007)	Link	1,009,290 bioactivities; 6,589 proteins; 427,325 small molecules.	Skaggs School of Pharmacy and Pharmaceutical Sciences	P
ChEMBL (Gaulton et al., 2012)	Link	12,077,491 bioactivities; 9,356 targets; 1,324,941 compounds (v17).	European Bioinformatics Institute (EBI)	P
DrugBank (Law et al., 2014)	Link	7,678 drugs (FDA-approved drugs, nutraceuticals, or experimental molecules); 4,270 proteins (v4).	Supported by The Metabolomics Innovation Centre and based at the University of Alberta	P
Drug-Gene Interaction Database (DGIdb) (Griffith et al., 2013)	Link	14,144 drug-gene interactions; 6,307 drugs; 2,611 genes.	Genome Institute at Washington University.	P
GOSTAR	Link	> 16 million bioactivities; > 6.3 million inhibitors;	GVK Bio	C
GPCR SARfari	Link	1,037,273 bioactivities; 147,292 GPCR ligands (v3).	EBI	P
Kinase SARfari	Link	532,155 bioactivities; 989 kinase domains; 45,189 kinase compounds (v6).	EBI	P
PDSP Ki Database (Roth et al., 2000)	Link	55,524 bioactivities; protein targets: receptors, neurotransmitter transporters, ion channels, enzymes	Funded by the NIMH Psychoactive Drug Screening Program and Heffter Research Institute and maintained by the University of North Carolina.	P
PubChem BioAssay (Wang et al., 2014)	Link	> 700,000 bioassays; hundreds of million bioactivities for small molecules and RNAi	National Center for Biotechnology Information (NCBI)	P
WOrld of Molecular BioAcTivity (WOMBAT) (Oprea, 2005)	Link	336,998 bioactivities; 1,966 unique targets (2013.1).	Sunset Molecular Discovery LLC	C
Compound-Indication				
MDL Drug Data Report (MDDR)	Link	150,000 compounds matched to pharmacological effects	Accelrys and Prous Science (Thomson Reuters)	C

Resource	Link	Data	Provider	Type
		Target-Pathway		
GeneGo Metabase	Link	> 1,400 pathway maps	Thomson Reuters	C
Kyoto Encyclopedia of Genes and Genomes (KEGG) (Kanehisa et al., 2014)	Link	> 450 pathway maps (v69.0)	Bioinformatics Center, Kyoto University and Human Genome Center, University of Tokyo,	P
Reactome (Croft et al., 2011)	Link	> 1,000 pathways	Ontario Institute for Cancer Research; Cold Spring Harbor Laboratory; New York University School of Medicine; EBI	P
WIKI	Link	1,797 pathways	Community-based	P
		Target-Disease		
Online Mendelian Inheritance in Man (OMIM) (Amberger et al., 2011)	Link	Genetic diseases and phenotypes linked (if possible) to relevant human genes.	Curated by John Hopkins University and hosted on the web by the NCBI.	P
Therapeutic Target Database (TTD) (Qin et al., 2014)	Link	2,360 protein and nucleic acid targets are linked to diseases	National University of Singapore	P
		Target-Function		
Gene Ontology (GO) (Blake et al., 2013)	Link	Gene products are related to molecular functions and biological processes through a defined ontology	Community-based	P

For a more structured overview, we have divided the listed databases into categories, e.g., DrugBank is reported as a database linking compounds (drugs) to targets. However, it should be noted that the reported databases often have multiple functionalities and allow for more applications than those described in the table. Reported numbers of compounds, proteins etc. are as of March 2014. The column "Type" reports whether the database is commercial (C) or public (P).

Supplemental references:

Amberger, J., Bocchini, C., and Hamosh, A. (2011). A new face and new challenges for Online Mendelian Inheritance in Man (OMIM®). Hum. Mutat. 32, 564–7.

Blake, J. A., Dolan, M., Drabkin, H., Hill, D. P., Li, N., Sitnikov, D., Bridges, S., Burgess, S., Buza, T., McCarthy, F., et al. (2013). Gene Ontology annotations and resources. Nucleic Acids Res. 41, D530–5.

Croft, D., O'Kelly, G., Wu, G., Haw, R., Gillespie, M., Matthews, L., Caudy, M., Garapati, P., Gopinath, G., Jassal, B., et al. (2011). Reactome: a database of reactions, pathways and biological processes. Nucleic Acids Res. 39, D691–7.

Gaulton, A., Bellis, L. J., Bento, A. P., Chambers, J., Davies, M., Hersey, A., Light, Y., McGlinchey, S., Michalovich, D., Al-Lazikani, B., et al. (2012). ChEMBL: a large-scale bioactivity database for drug discovery. Nucleic Acids Res. 40, D1100–7.

Griffith, M., Griffith, O. L., Coffman, A. C., Weible, J. V, McMichael, J. F., Spies, N. C., Koval, J., Das, I., Callaway, M. B., Eldred, J. M., et al. (2013). DGIdb: mining the druggable genome. Nat. Methods 10, 1209–10.

Kanehisa, M., Goto, S., Sato, Y., Kawashima, M., Furumichi, M., and Tanabe, M. (2014). Data, information, knowledge and principle: back to metabolism in KEGG. Nucleic Acids Res. 42, D199–205.

Law, V., Knox, C., Djoumbou, Y., Jewison, T., Guo, A. C., Liu, Y., Maciejewski, A., Arndt, D., Wilson, M., Neveu, V., et al. (2014). DrugBank 4.0: shedding new light on drug metabolism. Nucleic Acids Res. 42, D1091–7.

Liu, T., Lin, Y., Wen, X., Jorissen, R. N., and Gilson, M. K. (2007). BindingDB: a web-accessible database of experimentally determined protein-ligand binding affinities. Nucleic Acids Res. 35, D198–201.

Oprea, T. I. ed. (2005). Chemoinformatics in Drug Discovery. Weinheim, GER: Wiley-VCH Verlag GmbH & Co. KGaA.

Qin, C., Zhang, C., Zhu, F., Xu, F., Chen, S. Y., Zhang, P., Li, Y. H., Yang, S. Y., Wei, Y. Q., Tao, L., et al. (2014). Therapeutic target database update 2014: a resource for targeted therapeutics. Nucleic Acids Res. 42, D1118–23.

Roth, B. L., Lopez, E., Patel, S., and Kroeze, W. K. (2000). The Multiplicity of Serotonin Receptors: Uselessly Diverse Molecules or an Embarrassment of Riches? Neurosci. 6, 252–262.

Wang, Y., Suzek, T., Zhang, J., Wang, J., He, S., Cheng, T., Shoemaker, B. A., Gindulyte, A., and Bryant, S. H. (2014). PubChem BioAssay: 2014 update. Nucleic Acids Res. 42, D1075–82.
